# Kansas City Veterinary College

**Published:** 1900-05

**Authors:** 


### KANSAS CITY VETERINARY COLLEGE.

The ninth annual commencement and banquet of this college was held at the Midland Hotel, Kansas City, March 14, at 8 P.M. The degree of D.V.S. was conferred upon the following: Messrs. Robert H. Carswell, Edward Makins, Jr., Claude M. McFarland, Willis H. Meadors, David C. Moberly, Charles A. Mooney, Joseph W. Parker, Horace E. Rice; Drs. Wendell A. Knight, and William Folsetter, Drs. Knight and Folsetter having taken a post-graduate course.

The Faculty, graduates, alumni, and friends of the college gathered at the banquet table, and after the repast was served the Faculty address was delivered by Dr. I. J. Wolf, followed by presentation of the diplomas by Dr. C. J. Sihler; class response, by Dr. Joseph W. Parker. Other addresses were made, as follows: "Comparative Medicine," Dr. H. C. Babcock; "The Undergraduate," Mr. Arthur Trickett; "The Municipal Veterinarian," Hon. R. J. McFarland; "Veterinary Jurisprudence," J. A. McLane, Esq.; "The Veterinarian and the Breeder," Dr. W. Ross Cooper; "Veterinary Colleges," Dr. William Folsetter. The exercises proved a most pleasing arrangement. The graduating class is reported as having been exceptionally diligent throughout the session, and passed with high averages a rigid final examination.

